# Selected Moral Issues and the Stress Experienced by Paediatric Nurses

**DOI:** 10.3390/healthcare13111306

**Published:** 2025-05-30

**Authors:** Anna Stefanowicz-Bielska, Magdalena Słomion, Agnieszka Olińska, Małgorzata Rąpała, Julia Behling, Joanna Stefanowicz

**Affiliations:** 1 Division of Internal and Paediatric Nursing, Institute of Nursing and Midwifery, Faculty of Health Sciences, Institute of Maritime and Tropical Medicine, Medical University of Gdansk, 80-211 Gdansk, Poland; julia.behling@gumed.edu.pl; 2 Department of Paediatrics, Diabetology and Endocrinology, University Clinical Center of Gdansk, 80-211 Gdansk, Poland; 3 Department of Paediatrics, Nephrology and Hypertension, University Clinical Centre in Gdansk, 80-211 Gdansk, Poland; magdalena.slomion@gumed.edu.pl; 4 Prof. Dr Stanislaw Popowski Regional Specialized Children’s Hospital, 10-561 Olsztyn, Poland; agnieszka.olinska@onet.pl; 5 Department of Paediatric Surgery, Marciniak Hospital, 54-049 Wroclaw, Poland; mrapala@poczta.onet.pl; 6 Department of Pediatrics, Hematology and Oncology, University Clinical Center of Gdansk, 80-211 Gdansk, Poland; joanna.stefanowicz@gumed.edu.pl; 7 Department of Paediatrics, Haematology and Oncology, Faculty of Medicine, Medical University of Gdansk, 80-210 Gdansk, Poland; 8 Faculty of Health Sciences, Medical University of Gdansk, 80-210 Gdansk, Poland

**Keywords:** morals, issues, stress, nurses, child, parents

## Abstract

**Background/Objectives**: The high sensitivity of paediatric nurses directly influences the quality of nursing care provided to patients. The purpose of this study is to present the most frequent issues faced by paediatric nurses in their everyday work and their responses to difficult situations at work, define the actions applied when a difficult situation occurs, and assess the level of stress and other factors influencing the level of stress experienced by paediatric nurses. **Methods**: This study was conducted using an original survey form and a standardised psychological questionnaire based on the Perceived Stress Scale (PSS-10) for paediatric nurses. **Results**: The study involved 416 paediatric nurses and indicated a medium level of stress among the nurses. The median stress level, calculated as the sum of answers to questions based on the PSS-10, was 18 (16.0 ÷ 20.0), and the mean was 17.9 (min–max = 1–30). The median Sten score was 6 (5.0 ÷ 7.0), and the mean Sten score was 5.94 (min–max = 2–9). Nurses aged 21–30 years, who live in a city, have a Bachelor of Science in Nursing or a Master of Science in Nursing, and work ≥ 61 h a week and 161–250 h a month experience a higher level of stress. Factors such as choosing which child to help first, spending a great deal of time filling out medical documentation, obtaining a sick child’s consent to perform nursing procedures which the child does not understand, involving the minor in decision-making, impolite or offensive behaviour from a sick child or their parents, ineffective nursing and treatment methods, providing care against the opinion/views of a sick child or their parents, difficulties in or a lack of understanding of the situation of a sick child and their family, performing nursing procedures that can cause the child pain, and the inability to fulfil a sick child’s request increase the level of stress experienced by paediatric nurses. When a difficult situation occurs at work occurs, the nurses usually meet and talk about the situation with someone close (72.4%) or engage in other activities to avoid thinking about the situation (66.6%). They consult a psychologist/psychotherapist very rarely (9.6%) and a psychiatrist extremely rarely (4.6%). **Conclusions**: Polish paediatric nurses were found to experience a medium level of stress. Since paediatric nurses are exposed to stress, providing them with psychological care is important. The level of perceived stress is dependent on the nurse’s age, place of residence, and education, as well as weekly and monthly working durations. Paediatric nurses experience many difficult situations in their everyday work that influence their stress levels. Management should pay special attention to difficult workplace situations faced by paediatric nurses and implement regular actions to reduce the levels of stress experienced.

## 1. Introduction

The development of medical technologies has improved the quality of healthcare and provided therapeutic team members with greater control over the health and life of their patients; however, it has also contributed to various ethical and moral issues and intensified the level of stress experienced by medical personnel [1,2].

Ethics can be defined as the system of moral principles, values, and standards that influence a human being’s conduct, including losses and benefits, good and evil, and the determination of how these principles are reflected in individual conduct and group relationships [2,3].

The therapeutic team is responsible for ensuring a high quality of patient care. Nurses have very close contact with patients and their families. They witness the weakest and most sensitive, private, and personal moments in their patients’ lives. They are the first to notice feelings of fear, pain, sadness, loneliness, or despair in, of the patients and their family members. In addition, while performing their duties, nurses may be obliged to act on patients’ behalf and make decisions based on ethical judgement [2,4].

Children differ from adults in mental and social terms. The autonomy of children is the most important issue. Decisions on the acceptance of treatment and participation in diagnostic tests take the form of a trilateral relationship involving the “therapeutic team–the child–the parent/carer”. As the autonomy of children is limited, paediatric nurses must fulfil their duties with due regard for the child’s rights. A paediatric nurse should be able to prevent potential ethical conflicts by implementing efficient communication between parents and other therapeutic team members and be prepared to solve future problems [2,5].

Nurses must be able to manage various ethical issues regarding clinical conditions and make moral decisions. Adequate, mature moral sensitivity helps solve ethical dilemmas and justify actions. It also prevents ethical dilemmas and conflicts [2,6,7].

In addition, moral sensitivity is a basic characteristic that a nurse caring for patients, particularly children needing professional healthcare, must have [8]. The high sensitivity of paediatrics directly influences the quality of nursing care provided to patients [2].

Paediatric nurses encounter difficult situations that can cause moral anxiety and stress while performing their duties. In particular, nurses working in children’s intensive care units, as well as oncological, surgical, diabetes, and cardiological wards, are exposed to such circumstances.

### 1.1. Literature Review

Three databases were reviewed: PubMed, EBSCO, and Scopus. The following keywords were used: paediatric nurses, ethical dilemmas, stress, and moral sensitivity.

Previous research indicates that moral distress and ethical dilemmas are common among nurses working in paediatric settings [9,10,11,12,13,14,15].

The levels of moral distress and intercultural sensitivity are moderate, with evidence suggesting that higher moral distress may be associated with increased intercultural awareness [9].

In haematology and oncology units, certain clinical situations, although rare, have been reported to cause high levels of moral distress, especially in inpatient care. Nurses expressed a clear need for additional resources and ethics-focused training to better handle such scenarios [10].

In intensive care contexts, nurses’ attitudes toward the appropriateness of interventions, such as dialysis, were influenced by factors such as patient prognosis, quality of life, and comorbidities [11]. Experience in dialysis care also shaped how nurses valued family input and how likely they were to voice disagreement with clinical decisions [11]. Demographic and situational variables were found to play a key role in shaping professional beliefs and enhancing interprofessional communication [13].

### 1.2. Study Aim

The purpose of this study is to present difficult situations faced by paediatric nurses during their work, define the processes applied when such difficult situations occur, and assess the level of stress and the factors influencing the level of stress experienced by paediatric nurses.

Based on the overall objective of the research, the authors formulated the following research questions: What is the level of stress experienced by paediatric nurses? Does gender, age, place of residence, education, place of work, seniority in the profession, computer work time, work system, number of working hours per week and month, and form of employment affect the level of stress experienced by paediatric nurses? Does the level of stress affect the occurrence of additional diseases in paediatric nurses? How do certain situations affect the level of stress experienced by paediatric nurses? Does the method of handling difficult situation affect the level of stress experienced by paediatric nurses? Does a particular method of handling a difficult situation affect the level of stress experienced by paediatric nurses?

## 2. Materials and Methods

### 2.1. Design

A large-scale prospective survey was conducted from 1 March 2023 to 29 February 2024.

### 2.2. Participants

A total of 6 healthcare institutions were selected at random in the Pomeranian and Warmia-Masuria Voivodeship and invited to take part in this study. A total of 740 surveys were distributed, and 416 completed surveys were received. Study participants were active paediatric nurses. Nurses working in other wards and medical professions were excluded from the study.

### 2.3. Methods

This study involved a diagnostic survey that included an original survey form and a standardised Perceived Stress Scale-10 (PSS-10) questionnaire prepared by Sheldon Cohen, Tom Kamarck, and Robin Mermelstein (adopted to Polish conditions by Z. Juczyńskiego and N. Ogińska-Bulik) [16,17].

#### 2.3.1. Original Survey Form

The first page of the survey form included information about and an invitation to participate in this study.

The survey form consisted of questions on sociodemographics (sex, age, place of residence, education, workplace, work experience), amount of time spent working on computers while on duty, length of shifts (8/12 h), day/night shift, work during holidays, number of working hours per week/month, form of employment, chronic diseases.

#### 2.3.2. Perceived Stress Scale (PSS-10)

PSS-10 is used to measure perceived stress. It contains 10 questions concerning subjective feelings related to personal issues and events, conduct, and handling methods. A respondent answers by entering a relevant value, i.e., 0—never, 1—almost never, 2—sometimes, 3—fairly often, 4—very often. Internal consistency in the survey was verified with a group of 120 adults, and Cronbach’s alpha was 0.86. In the original version, the internal reliability of the scale, as assessed based on Cronbach’s alpha, is from 0.84 to 0.86 for three samples studied by Cohen and partners (1983) [16,17].

Before a general perceived stress intensity ratio is calculated, it is necessary to change the scoring for answers to positive questions, i.e., 4, 5, 7, and 8, based on the following rule: 0 = 4, 1 = 3, 3 = 1, 4 = 0. The scoring for answers to questions 1, 2, 3, 6, 9, and 10 is not changed. The general scale outcome is the sum of all scores, whose theoretical distribution is from 0 to 40; the greater the score, the greater the perceived stress. The general ratio, when converted into standardised units, is subject to interpretation and adjusted to the characteristics of the Sten scale. A score of 1 to 4 Sten is treated as low, while a score of 7 to 10 is treated as high. A score of 5 or 6 is treated as average. The scale result reflects the general assessment of mental comfort connected with problem handling. A high PSS-10 ratio is associated with the existence of various mental and somatic symptoms, which is why it is a measure of chronic stress, a risk factor for various diseases [16,17].

The paper version of PSS-10 was used with the consent of the Psychological Test Laboratory of the Polish Psychological Society.

#### 2.3.3. Data Collection

The data were collected in writing from 1 April 2023 to 29 February 2024 using a survey form and a psychological questionnaire. The survey forms and questionnaires were delivered to all nurses via the heads of selected healthcare institutions. Participation in this study was voluntary. The study was conducted after receiving consent from the heads of particular healthcare institutions and the approval of the Independent Bioethical Commission for Scientific Studies at the Medical University of Gdansk (NKBBN/91/2023).

#### 2.3.4. Statistical Analysis

The results were subjected to statistical processing. The number of cases (N), mean, standard deviation (SD), median, range (min–max), and lower and upper quartiles (25 Q–75 Q) of the quantitative parameters were calculated for all groups.

Depending on the distribution, quantitative data were presented as follows:

1. Mean ±SD in the case of variables with normal distribution.
2. Median and interquartile range M (25 Q ÷ 75 Q) for variables with non-normal distribution.

Qualitative variables were presented as absolute values and percentages (%).

The normality of the distribution was tested using the Shapiro–Wilk test, and homogeneity of variance was checked using Levene’s test.

The hypothesis of equality of mean parameters in independent groups with homogeneous variance was verified using one-way analysis of variance (ANOVA), or for groups with heterogeneous variance, using the non-parametric Mann–Whitney U test (for two groups) and the Kruskal–Wallis test (for three or more groups).

A *p*-value of less than 0.05 was required to reject the null hypothesis. Statistical analysis was performed using the computer statistical software package Statistica Ver. 13.3. (TIBCO Software Inc., Corporate headquarters, 2623 Camino Ramon, Suite 200, San Ramon, CA, USA).

## 3. Results

### 3.1. Characterisation of Nurses

This study involved 416 paediatric nurses. Most nurses were women aged 51–60 years, living in a city, with a Master of Science in Nursing (MN), ≥21 years of professional experience in nursing, and working 161 to 200 h a month in the 12 h shift system—day/night and during holidays—based on an employment contract (Table 1).

### 3.2. Level of Stress and the Impact of Various Factors on the Level of Stress Experienced by Paediatric Nurses

The median stress experienced by paediatric nurses was 18 (16.0 ÷ 20.0) and the mean was 17.9 (min–max = 1–30); the sum of the answers to the PSS-10 questions and the median of Sten scores was 6 (5.0 ÷ 7.0), while the mean of Sten scores was 5.94 (min–max = 2–9).

Nurses aged 21–30 years who live in a city, have a BN or an MN in nursing, and work ≥ 61 h a week and 161–250 h a month experience a higher level of stress (Table 1). This study’s participants suffered mainly from obesity, overweight, hypertension, depression, and neurosis (Table 2). Seven participants were diagnosed with eating disorders: four had anorexia and three had bulimia.

This study did not analyse the relationship between the existence of selected chronic diseases and the level of stress experienced by Polish paediatric nurses (Table 2).

### 3.3. Impact of Difficult Situations on the Level of Stress Experienced by Paediatric Nurses

There were significant differences in anxiety levels between paediatric nurses who experienced specific difficult situations at work and those who did not (Table 3).

### 3.4. Conduct Following the Occurrence of a Difficult Situation and the Level of Stress Experienced by Paediatric Nurses

The moment a difficult situation occurs, most of paediatric nurses usually obey the rules set out in the code of ethics for nurses (409/415; 98.6%), rules set out in medical procedures applicable to their healthcare institution (408/415; 98.3%), as well as legal regulations (411/416; 98.8%).

There were no significant differences in anxiety levels among paediatric nurses who used specific treatments during difficult situations at work and those who did not (Table 4).

### 3.5. State and Conduct (Response to a Situation) Following the Occurrence of a Difficult Situation and the Level of Stress Experienced by Paediatric Nurses

There were significant differences in anxiety levels between paediatric nurses who had a specific response to a difficult situation and those who did not (Table 5).

## 4. Discussion

The nursing of children is associated with many significant ethical and moral challenges. Paediatric nurses are commonly perceived as professionals with relevant knowledge of children's nursing.

During their everyday work, paediatric nurses experience various difficult situations where they must show empathy and be capable of deep reflection and involvement in important moral issues [18,19].

Such experiences may include moral uncertainty when nurses and other specialists are not clear about what actions should be taken in a specific situation, as well as moral suffering when nurses feel that they cannot, for various reasons, take actions they find ethically correct [19]. Morally difficult situations encountered while taking care of very sick children can result in nurses suffering moral anxiety and could be the source of their moral stress [20].

Moral stress is a very common experience among nurses and one of the major reasons for dissatisfaction with work, burnout, and resignation [20,21].

Among all groups of nurses, paediatric nurses are especially exposed to stress and burnout. As stress factors, the literature usually mentions a large workload [21,22], an increased number of patients [21,23], human resources shortages [21,24], and shift work where working time exceeds eight hours a day [21,25].

The above factors contribute to emotional and mental exhaustion and have a significant impact on the health of paediatric nurses [21,26].

The purpose of our survey was to assess the level of stress, analyse difficult situations faced by paediatric nurses during their work, define the actions to be applied when such difficult situations occur, and assess various factors that influence the level of stress experienced by paediatric nurses.

The survey involved 416 paediatric nurses. Most of the nurses were women aged 51–60 years, living in a city, with an MN in nursing and ≥21 years of professional experience in nursing, working the 12 h shift system (day/night) and during holidays. The nurses work mainly on the basis of employment contracts ≤40 h a week and ≥ 251 h a month.

The most frequent issues faced by the nurses during their everyday work include the need to keep very extensive medical documentation (spending a great deal of time filling out medical documentation reduces time spent with a sick child and their parents), experiencing impolite or offensive behaviour from a sick child or their parents (aggression by a child and/or their parents), performing work despite an insufficient number of nurses and increased workload, performing nursing procedures which can cause pain to the child, and the inability to fulfil sick children’s requests.

When a difficult situation occurs, paediatric nurses usually obey the rules set out in the code of ethics for nurses, rules set out in medical procedures applicable to their healthcare institution, and legal regulations.

Following a difficult situation, paediatric nurses usually meet to talk about the situation with someone close, carry out other activities to avoid thinking about the situation, and think about and analyse the situation multiple times.

Using the paediatric version of the Moral Distress Scale—Revised (MDS-R) questionnaire, Iranian researchers assessed the moral distress experienced by nurses in selected paediatric hospitals in Tehran. The study involved 195 paediatric nurses working at three selected specialised paediatric university hospitals in Tehran. The mean and standard deviation of the total result of moral distress were 106.41 ± 61.64 (range 10–257). The situation that involved the greatest moral suffering was “observing students of medicine applying painful treatment methods to patients only to learn certain skills”. The total result of moral distress is substantially higher for paediatric nurses, and nurses with a master’s degree experienced greater moral distress than nurses with a bachelor’s degree [20].

In our study, the level of stress experienced by paediatric nurses was moderately high, with a median PSS-10 score of 18 (IQR: 16.0–20.0) and a Sten score of 6 (IQR: 5.0–7.0). Higher stress levels were particularly evident among nurses aged 21–30, those living in urban areas, holding a master’s degree in nursing, and working ≥ 61 h per week or 161–250 h per month. Several specific work-related situations significantly contributed to elevated stress levels, including ethical and organisational challenges such as deciding which child to prioritise, extensive documentation duties, lack of parental presence during treatment, obtaining consent from children who do not understand procedures, impolite behaviour from patients or their families, and performing painful or morally challenging procedures.

These findings are consistent with previous studies that have highlighted the psychological and ethical burden of paediatric nursing. For example, prior work has shown that secondary traumatic stress is prevalent among paediatric nurses, independent of age or years of experience, and that nurses use a range of coping mechanisms from emotional support to behavioural disengagement [27].

Other research has emphasised that ethical challenges are a major source of stress, with moral sensitivity often increasing with age and experience. However, this does not always reduce stress levels [2,28]. Qualitative studies have further supported these insights, revealing emotional fatigue, communication barriers, and frustration in ethically complex care situations [13, 19].

Consistent with our results, studies from various healthcare systems have confirmed that factors such as workload, clinical department, and coping strategies significantly influence stress levels [27,29]. Importantly, moral resistance has been identified as a mediating factor between moral suffering and professional identity [30].

Our study contributes to the growing body of literature by identifying specific ethical and organisational stressors in paediatric nursing and underlining the need for institutional mechanisms that support ethical reflection, psychological assistance, and workload management, especially for younger or less experienced nurses.

It is worth pointing out that the study has indicated that when a difficult situation occurs at work, Polish paediatric nurses usually meet and talk about the situation with someone close (72.4%), perform other activities to avoid thinking about the situation (66.6%), or pray (43%); however, very few consult a psychologist/psychotherapist (9.6%) or a psychiatrist (4.6%).

Up to 40.3% of the nurses transfer their problems at work to their personal lives, and 32.8% transfer their problems to their family members. Although 51.8% have a guilty conscience and blame themselves, only 6.7% take substances and 13.5% drink alcohol.

The results presented herein form a special challenge for the management of nursing teams and should be used as input for developing conduct guidelines to protect Polish paediatric nurses against the consequences of stress.

The limitations of this study are as follows:

- Most of the participants were female, which made it impossible to prove the impact of gender.
- There was a relatively small number of respondents in relation to the total number of active paediatric nurses in Poland.
- The impact of the workplace, i.e., the type of ward, could not be proven.

## 5. Conclusions

Polish paediatric nurses experience a medium level of stress. Since paediatric nurses are exposed to stress, they should be provided with psychological care. The level of perceived stress is dependent on the nurse’s age, place of residence, education, and weekly and monthly working times. Paediatric nurses experience many difficult situations in their everyday work that influence their stress levels. Management should pay special attention to the difficult situations faced by paediatric nurses when performing their duties and implement regular actions to reduce the levels of stress that they experience.

## Figures and Tables

**Table 1:** Sociodemographic data and the level of stress experienced by paediatric nurses.

Sociodemographic Data	Answers	N (%)	Sum of Answers to PSS-10 Questions	Sten Score
sex	male	14 (3.4%)	17.5 (15.0 ÷ 19.0)	6 (5.0 ÷ 6.0)
	female	402 (96.6%)	18 (16.0 ÷ 20.0)	6 (5.0 ÷ 7.0)
	*p*		0.212 *	0.266 *
age	21–30	94 (22.6%)	18.5 ± 2.5	6.13 ± 0.86
	31–40	48 (11.5%)	17.5 ± 2.3	5.79 ± 0.74
	41–50	91 (21.9%)	18.0 ± 2.8	5.96 ± 0.91
	51–60	153 (36.8%)	17.8 ± 3.4	5.93 ± 1.06
	≥61	30 (7.2%)	16.3 ± 4.4	5.57 ± 1.19
	*p*		0.013 ** 21–30 vs. ≥61—*p* = 0.02 ****	0.056 **
place of residence	city	336 (80.7%)	18.0 ± 3.0	5.99 ± 0.95
	village	80 (19.3%)	17.1 ± 3.5	5.74 ± 1.02
	*p*		0.015 **	0.037 **
nursing education	S	139 (33.4%)	17.3 ± 3.5	6 (5.0 ÷ 7.0)
	BN	121 (29.1%)	18.2 ± 2.8	6 (5.0 ÷ 7.0)
	MN	156 (37.5%)	18.1 ± 2.8	6 (5.0 ÷ 7.0)
	*p*		0.018 ** S vs. BN—*p* = 0.04 **** S vs. MN—*p* = 0.05 ****	0.185 **
workplace	group 1	173 (41.6%)	18.0 ± 3.1	6 (5.0 ÷ 7.0)
	group 2	48 (11.5%)	17.0 ± 3.6	6 (5.0 ÷ 6.0)
	group 3	82 (19.7%)	18.3 ± 3.3	6 (5.0 ÷ 7.0)
	group 4	39 (9.4%)	17.5 ± 3.4	6 (5.0 ÷ 6.0)
	group 5	28 (6.7%)	17.9 ± 1.6	6 (5.0 ÷ 6.0)
	group 6	46 (11.1%)	17.7 ± 2.3	6 (5.0 ÷ 6.0)
	*p*		0.240 **	0.254 ***
work experience in nursing	≤5 years	95 (22.9%)	18.5 ± 2.5	6.14 ± 0.83
	6–10 years	36 (8.7%)	17.2 ± 2.3	5.78 ± 0.76
	11–20 years	32 (7.8%)	17.4 ± 2.2	5.69 ± 0.78
	≥21 years	251 (60.6%)	17.8 ± 3.4	5.92 ± 1.05
	*p*		0.072 **	0.064 **
time spent on computer work	≤2 h	65 (15.7%)	18 (16.0 ÷ 19.0)	6 (5.0 ÷ 6.0)
	3–4 h	223 (53.6%)	18 (16.0 ÷ 19.0)	6 (5.0 ÷ 6.0)
	5–6 h	78 (19%)	18.0 (16.0 ÷ 21.0)	6 (5.0 ÷ 7.0)
	≥7 h	48 (11.7%)	19 (16.0 ÷ 20.5)	6 (5.0 ÷ 7.0)
	*p*		0.269 ***	0.275 ***
working system	12 h	331 (79.9%)	17.8 ± 3.0	5.91 ± 0.94
	8 h	83 (20.1%)	18.2 ± 3.5	6.07 ± 1.06
	*p*		0.352 **	0.179 **
night shift	yes	323 (77.6%)	17.8 ± 2.99	5.92 ± 0.95
	no	93 (22.4%)	18.1 ± 3.4	6.02 ± 1.04
	*p*		0.494 **	0.357 **
work during holidays	yes	333 (80%)	17.8 ± 2.95	5.92 ± 0.94
	no	83 (20%)	18.1 ± 3.6	6.04 ± 1.08
	*p*		0.461 **	0.312 **
number of working hours per week	≤40 h	187 (45.1%)	17.6 ± 3.2	5.87 ± 0.97
	41–50 h	153 (36.9%)	17.9 ± 3.0	5.93 ± 0.96
	51–60 h	44 (10.6%)	17.9 ± 2.8	5.93 ± 0.93
	≥61 h	31 (7.4%)	19.3 ± 2.8	6.39 ± 0.99
	*p*		0.043 ** ≤40 vs. ≥61 h—*p* = 0.04 ****	0.052 **
number of working hours per month	≤160 h	146 (35.3%)	17.5 (16.0 ÷ 19.0)	6 (5.0 ÷ 6.0)
	161–200 h	208 (50.2%)	18 (16.0 ÷ 20.0)	6 (5.0 ÷ 7.0)
	201–250 h	39 (9.4%)	18 (16.0 ÷ 20.0)	6 (5.0 ÷ 7.0)
	≥251 h	21 (5.1%)	18 (17.0 ÷ 21.0)	6 (6.0 ÷ 7.0)
	*p*		0.039 *** ≤160 vs. 161–200 h—*p* = 0.08 ****	0.033 *** ≤160 vs. 161–200 h—*p* = 0.08 ****
form of employment	civil-law agreement	96 (23.1%)	17.5 ± 3.6	5.81 ± 1.09
	employment contract	320 (76.9%)	18.0 ± 2.9	5.98 ± 0.93
	*p*		0.205 **	0.141 **

Group 1: paediatric department, infectious paediatric diseases department, primary healthcare clinic, specialised clinic, hospital emergency department/night emergency department, admission room, emergency department; Group 2: paediatric gastroenterology department, paediatric nephrology department, paediatric diabetology department, paediatric neurology department, paediatric psychiatry department, paediatric cardiology department; Group 3: paediatric orthopaedics department, paediatric laryngology department, paediatric surgery department; Group 4: neonatology department, infant pathology department; Group 5: infant intensive care department, paediatric intensive care department; Group 6: paediatric hospice, paediatric haematology and oncology department; S—secondary; BN—Bachelor of Science in Nursing; MN—Master of Science in Nursing; * Mann–Whitney U test; ** ANOVA test; *** Kruskal–Wallis test; **** analysis post hoc—Scheffe’s test.

**Table 2:** Chronic diseases and the level of stress experienced by paediatric nurses.

Chronic Disease	Answers	N (%)	Sum of Answers to PSS-10 Questions	Sten Score
obesity	yes	41 (9.9%)	17.8 ± 3.6	5.95 ± 1.09
	no	375 (90.1%)	17.9 ± 3.0	5.94 ± 0.96
	*p*		0.820 **	0.937 **
overweight	yes	117 (28.2%)	18.3 ± 2.9	6.06 ± 0.99
	no	298 (71.8%)	17.7 ± 3.1	5.90 ± 0.96
	*p*		0.083 **	0.128 **
hypertension	yes	79 (19%)	17.7 ± 3.7	5.89 ± 1.09
	no	337 (81%)	17.9 ± 2.9	5.95 ± 0.94
	*p*		0.518 **	0.584 **
depression	yes	25 (6%)	19 (17.0 ÷ 20.0)	6 (6.0 ÷ 7.0)
	no	391 (94%)	18 (16.0 ÷ 20.0)	6 (5.0 ÷ 7.0)
	*p*		0.576 *	0.512 *
neurosis	yes	21 (5.1%)	18 (15.0 ÷ 20.0)	6 (5.0 ÷ 7.0)
	no	394 (94.9%)	18 (16.0 ÷ 20.0)	6 (5.0 ÷ 7.0)
	*p*		0.541 *	0.710 *
other chronic diseases	yes	123 (29.7%)	17.9 ± 2.8	5.90 ± 0.94
	no	291 (70.3%)	17.9 ± 3.2	5.96 ± 0.98
	*p*		0.979 **	0.612 **

* Mann–Whitney U test; ** ANOVA test.

**Table 3:** Occurrence of difficult situations and the level of stress experienced by paediatric nurses.

Difficult Situation	Answers	N (%)	Sum of Answers to PSS-10 Questions	Sten Score
starting cardio-pulmonary resuscitation of a sick child	yes	57 (13.8%)	17.9 ± 2.2	5.91 ± 0.79
	no	356 (86.2%)	17.8 ± 3.2	5.94 ± 1.00
	*p*		0.808 **	0.820 **
stopping cardio-pulmonary resuscitation of a sick child	yes	16 (3.9%)	18.5 (16.0 ÷ 20.0)	6 (5.0 ÷ 6.5)
	no	393 (96.1%)	18 (16.0 ÷ 20.0)	6 (5.0 ÷ 7.0)
	*p*		0.531 *	0.755 *
selecting which child to help first	yes	110 (26.6%)	18.4 ± 2.8	6.13 ± 0.96
	no	304 (73.4%)	17.7 ± 3.2	5.88 ± 0.97
	*p*		0.024 **	0.019 **
spending a great deal of time filling in medical documentation and not with a sick child and their parents	yes	245 (59.2%)	18.3 ± 2.8	6.05 ± 0.94
	no	169 (40.8%)	17.3 ± 3.4	5.78 ± 0.99
	*p*		0.001 **	0.004 **
obtaining a sick child’s consent to perform nursing procedures that the child does not understand	yes	186 (45.3%)	18.3 ± 2.6	6.08 ± 0.89
	no	225 (54.7%)	17.5 ± 3.4	5.83 ± 1.02
	*p*		0.004 **	0.008 **
obtaining a sick child’s parent’s consent to perform nursing procedures that the parent does not understand	yes	202 (49%)	18.1 ± 2.7	6.02 ± 0.91
	no	210 (51%)	17.6 ± 3.4	5.85 ± 1.02
	*p*		0.062 **	0.064 **
providing inadequate care and treatment	yes	57 (13.8%)	18.4 ± 3.0	6.07 ± 1.07
	no	355 (86.2%)	17.8 ± 3.1	5.92 ± 0.95
	*p*		0.159 **	0.263 **
involving the minor in decision-making	yes	159 (38.5%)	18.3 ± 2.8	6.06 ± 0.93
	no	254 (61.5%)	17.6 ± 3.2	5.86 ± 0.99
	*p*		0.013 **	0.040 **
dealing with impolite or offensive behaviour by a sick child or their parents	yes	266 (64.1%)	18.2 ± 3.1	6.05 ± 0.99
	no	149 (35.9%)	17.2 ± 3.1	5.75 ± 0.91
	*p*		0.001 **	0.003 **
extending the process of dying by persistent therapy	yes	56 (13.5%)	17.8 ± 3.7	5.93 ± 1.13
	no	359 (86.5%)	17.9 ± 3.0	5.94 ± 0.95
	*p*		0.810 **	0.926 **
accelerating the process of dying by inadequate care	yes	9 (2.2%)	17 (16.0 ÷ 21.0)	6 (5.0 ÷ 7.0)
	no	406 (97.8%)	18 (16.0 ÷ 20.0)	6 (5.0 ÷ 7.0)
	*p*		0.920 *	0.953 *
ineffective nursing and treatment methods	yes	142 (34.3%)	18.5 ± 2.8	6.12 ± 0.92
	no	272 (65.7%)	17.5 ± 3.2	5.84 ± 0.98
	*p*		0.001 **	0.005 **
care inconsistent with the opinion/views of a sick child or their parents	yes	88 (21.2%)	18.5 ± 3.5	6.13 ± 1.07
	no	326 (78.8%)	17.7 ± 3.0	5.89 ± 0.94
	*p*		0.036 **	0.040 **
a parent giving up modern nursing and treatment methods owing to financial problems	yes	46 (11.1%)	18.5 (16.0 ÷ 20.0)	6 (5.0 ÷ 7.0)
	no	367 (88.9%)	18 (16.0 ÷ 20.0)	6 (5.0 ÷ 7.0)
	*p*		0.171 *	0.104 *
providing insufficient pain relief treatment	yes	92 (22.2%)	18.4 ± 3.0	6.09 ± 1.00
	no	320 (77.8%)	17.7 ± 3.1	5.90 ± 0.96
	*p*		0.063 **	0.098 **
giving false hope to patients, parents, and families	yes	48 (11.6%)	18.1 ± 3.8	6.00 ± 1.11
	no	365 (88.4%)	17.8 ± 3.0	5.93 ± 0.95
	*p*		0.642 **	0.660 **
difficulties and a lack of understanding of the situation of a sick child and their family	yes	125 (30.2%)	18.6 ± 3.1	6.15 ± 1.00
	no	289 (69.8%)	17.6 ± 3.0	5.85 ± 0.94
	*p*		0.002 **	0.003 **
doubts related to a nursing and treatment method	yes	154 (37.4%)	18.2 ± 3.3	6.03 ± 1.05
	no	258 (62.6%)	17.7 ± 2.9	5.89 ± 0.92
	*p*		0.102 **	0.175 **
a need to report the incompetence of a nurse or a doctor	yes	53 (12.8%)	18.3 ± 2.6	6.08 ± 0.92
	no	361 (87.2%)	17.8 ± 3.2	5.92 ± 0.98
	*p*		0.317 **	0.284 **
being exposed to the impolite or offensive behaviour of a patient or their family while taking care of a sick child	yes	258 (62.5%)	18.3 ± 2.9	6 (6.0 ÷ 7.0)
	no	155 (37.5%)	17.1 ± 3.2	6 (5.0 ÷ 6.0)
	*p*		*p* < 0.001 **	*p* < 0.001 *
performing work despite an insufficient number of nurses and an increased workload	yes	276 (66.7%)	18.1 ± 3.0	5.99 ± 0.97
	no	138 (33.3%)	17.5 ± 3.3	5.84 ± 0.96
	*p*		0.070 **	0.133 **
cooperation with nurses who perform their duties in an incompetent way	yes	126 (30.4%)	18.2 ± 3.0	6.03 ± 1.0
	no	288 (69.6%)	17.7 ± 3.1	5.90 ± 0.96
	*p*		0.149 **	0.214 **
acting for the good of a child but in non-conformity with the dictates of conscience/belief of the sick child or their parents	yes	48 (11.6%)	18.2 ± 3.9	6.04 ± 1.11
	no	365 (88.4%)	17.8 ± 3.0	5.93 ± 0.95
	*p*		0.483 **	0.460 **
acting against one’s own views and conscience	yes	43 (10.4%)	17.8 ± 2.9	5.90 ± 1.02
	no	369 (89.6%)	17.9 ± 3.1	5.94 ± 0.97
	*p*		0.915 **	0.818 **
performing nursing procedures using inadequate equipment owing to a lack of adequate equipment at work	yes	64 (15.5%)	17.6 ± 3.9	5.86 ± 1.17
	no	348 (84.5%)	17.9 ± 2.9	5.96 ± 0.93
	*p*		0.374 **	0.435 **
a need to take care of a sick child without compliance with personal dignity and intimacy rules	yes	32 (7.7%)	17.3 ± 3.3	5.81 ± 1.06
	no	381 (92.3%)	17.9 ± 3.1	5.96 ± 0.96
	*p*		0.313 **	0.424 **
performing nursing procedures that can cause pain to the child	yes	277 (67.4%)	18 (16.0 ÷ 20.0)	6 (5.0 ÷ 7.0)
	no	134 (32.6%)	17 (16.0 ÷ 19.0)	6 (5.0 ÷ 6.0)
	*p*		0.003 *	0.015 *
contact with a child or their parent who refuses further treatment	yes	145 (35.1%)	18.2 ± 3.3	6.05 ± 1.06
	no	268 (64.9%)	17.7 ± 3.0	5.89 ± 0.91
	*p*		0.149 **	0.109 **
inability to fulfil sick children’s requests	yes	212 (51.7%)	18.3 ± 2.8	6.05 ± 0.93
	no	198 (48.3%)	17.4 ± 3.3	5.82 ± 0.99
	*p*		0.002 **	0.016 **

* Mann–Whitney U test; ** ANOVA test.

**Table 4:** Conduct following the occurrence of a difficult situation and the level of stress experienced by paediatric nurses.

Conduct Following the Occurrence of a Difficult Situation	Answers	N (%)	Sum of Answers to PSS-10 Questions	Sten Score
I act in conformity with the dictates of my conscience	yes	247 (81%)	17.8 ± 3.0	5.92 ± 0.95
	no	58 (19%)	18.3 ± 3.6	6.10 ± 1.07
	*p*		0.203 **	0.172 **
I act in line with religious principles/values	yes	308 (74.2%)	18 (16.0 ÷ 20.0)	6 (5.0 ÷ 7.0)
	no	107 (25.8%)	18 (17.0 ÷ 20.0)	6 (6.0 ÷ 7.0)
	*p*		0.102 *	0.190 *
I follow my superior’s opinion	yes	358 (86.3%)	17.9 ± 3.1	5.95 ± 0.97
	no	57 (13.7%)	17.6 ± 3.2	5.86 ± 0.99
	*p*		0.429 **	0.503 **
I follow my colleagues’ opinions	yes	289 (69.5%)	17.8 ± 3.2	5.91 ± 0.99
	no	127 (30.5%)	18.1 ± 2.8	6.00 ± 0.93
	*p*		0.306 **	0.402 **

* Mann–Whitney U test; ** ANOVA test.

**Table 5:** State and conduct (response to a situation) following the occurrence of a difficult situation and the level of stress experienced by paediatric nurses.

Conduct Following the Occurrence of a Difficult Situation	Answers	N (%)	Sum of Answers to PSS-10 Questions	Sten Score
I transfer professional problems to my personal life	yes	167 (40.3%)	18.4 ± 2.7	6.10 ± 0.92
	no	247 (59.7%)	17.5 ± 3.3	5.83 ± 0.99
	*p*		0.007 **	0.005 **
I transfer professional problems onto my family members	yes	136 (32.8%)	18.3 ± 2.8	6.07 ± 0.95
	no	279 (67.2%)	17.7 ± 3.2	5.87 ± 0.98
	*p*		0.049 **	0.049 **
I have a guilty conscience/I blame myself	yes	215 (51.8%)	18 (16.0 ÷ 20.0)	6 (5.0 ÷ 7.0)
	no	200 (48.2%)	18 (16.0 ÷ 20.0)	6 (5.0 ÷ 7.0)
	*p*		0.146 *	0.246 *
I eat more	yes	125 (30.1%)	18.2 ± 2.6	6.02 ± 0.90
	no	290 (69.9%)	17.7 ± 3.3	5.90 ± 1.00
	*p*		0.142 **	0.246 **
I avoid eating	yes	64 (15.5%)	17.9 ± 3.1	5.91 ± 1.03
	no	349 (84.5%)	17.7 ± 3.1	5.95 ± 0.96
	*p*		0.898 **	0.733 **
I smoke cigarettes	yes	87 (21%)	18.2 ± 2.9	6.03 ± 0.96
	no	328 (79%)	17.8 ± 3.1	5.91 ± 0.97
	*p*		0.290 **	0.306 **
I drink alcohol	yes	56 (13.5%)	18 (15.0 ÷ 20.0)	6 (5.0 ÷ 7.0)
	no	358 (86.5%)	18 (16.0 ÷ 20.0)	6 (5.0 ÷ 7.0)
	*p*		0.567 *	0.216 *
I meet to talk about the difficult situation with someone close	yes	301 (72.4%)	18.1 ± 2.8	6.00 ± 0.93
	no	115 (27.6%)	17.2 ± 3.7	5.78 ± 1.05
	*p*		0.003 **	0.04 **
I pray	yes	179 (43%)	17.6 ± 3.5	5.88 ± 1.02
	no	237 (57%)	18.1 ± 2.8	5.98 ± 0.93
	*p*		0.140 **	0.296 **
I consult a psychologist/psychotherapist	yes	40 (9.6%)	17.3 ± 3.4	5.75 ± 0.95
	no	376 (90.4%)	17.9 ± 3.1	5.96 ± 0.97
	*p*		0.189 **	0.193 **
I consult a psychiatrist	yes	19 (4.6%)	19 (16.0 ÷ 21.0)	6 (5.0 ÷ 7.0)
	no	397 (95.4%)	18 (16.0 ÷ 20.0)	6 (5.0 ÷ 7.0)
	*p*		0.528 *	0.827 *
I take substances	yes	28 (6.7%)	18.1 ± 3.3	6.00 ± 1.00
	no	387 (93.3%)	17.8 ± 3.1	5.93 ± 0.97
	*p*		0.614 **	0.588 **
I carry out other activities so as not to think about the situation	yes	277 (66.6%)	18 (17.0 ÷ 20.0)	6 (6.0 ÷ 7.0)
	no	139 (33.4%)	17 (16.0 ÷ 19.0)	6 (5.0 ÷ 6.0)
	*p*		*p* < 0.001 *	*p* < 0.001 *
I do physical exercise/practise sports	yes	192 (46.2%)	18.1 ± 2.9	6.01 ± 0.95
	no	224 (53.8%)	17.7 ± 3.2	5.88 ± 0.98
	*p*		0.154 **	0.203 **
I think about and analyse the difficult situation many times	yes	303 (72.8%)	18 (16.0 ÷ 20.0)	6 (5.0 ÷ 7.0)
	no	113 (27.2%)	18 (16.0 ÷ 20.0)	6 (5.0 ÷ 7.0)
	*p*		0.300 *	0.402 *

* Mann–Whitney U test; ** ANOVA test.

## Data Availability

Data are available on request from the corresponding author.

## Abbreviations

The following abbreviations are used in this manuscript:

PSS-10	Perceived Stress Scale
N	number of cases
SD	standard deviation
MN	Master of Science in Nursing
BN	Bachelor of Science in Nursing
S	secondary
MDS-R	Moral Distress Scale—Revised
